# Cochlear Implantation Improves Both Speech Perception and Patient-Reported Outcomes: A Prospective Follow-Up Study of Treatment Benefits among Adult Cochlear Implant Recipients

**DOI:** 10.3390/jcm11082257

**Published:** 2022-04-18

**Authors:** Kasper Møller Boje Rasmussen, Niels Cramer West, Michael Bille, Matilde Grønborg Sandvej, Per Cayé-Thomasen

**Affiliations:** 1 Department of Otorhinolaryngology, Head and Neck Surgery and Audiology, Rigshospitalet University Hospital of Copenhagen, 2100 Copenhagen, Denmark; niels.cramer.west@regionh.dk (N.C.W.); michael.bille@regionh.dk (M.B.); matilde.groenborg.sandvej@regionh.dk (M.G.S.); per.caye-thomasen.01@regionh.dk (P.C.-T.); 2 Faculty of Health and Medical Sciences, University of Copenhagen, 2200 Copenhagen, Denmark

**Keywords:** treatment, hearing rehabilitation, hearing loss, outcome, patient-reported outcome measures, PROM, cochlear implant

## Abstract

Cochlear implantation is considered the best treatment option for patients with severe-to-profound sensorineural hearing loss for whom conventional hearing aids are insufficient. We used a repeated measures longitudinal approach to evaluate speech recognition and patient-reported outcomes after cochlear implantation in an unbiased cohort of Danish adult patients in a prospective cohort study. We assessed 39 recipients before and two times after implantation using a battery of tests that included Dantale I, the Danish Hearing in Noise Test, the Nijmegen Cochlear Implant Questionnaire, and the Speech, Spatial, and Qualities of Hearing Scale. The study group improved significantly on all outcome measures following implantation. On average, Dantale I scores improved by 29 percentage points and Hearing in Noise Test scores improved by 22 percentage points. Most notably, the average Dantale score improved from 26 to 70% in the CI in quiet condition and from 12 to 42% in the cochlear implantation in noise condition when tested monaurally. Dantale demonstrated a significant positive correlation with Nijmegen Cochlear Implant Questionnaire and Speech, Spatial, and Qualities of Hearing Scale scores, while Hearing in Noise Test had no significant correlation with the patient-reported outcome measures. Patients improved significantly at 4 months and marginally improved further at 14 months, indicating that they were approaching a plateau. Our study’s use of audiometric and patient-reported outcome measures provides evidence of the treatment benefits of cochlear implantation in adults, which may help physicians advise patients on treatment decisions and align treatment benefit expectations, as well as serve as a foundation for the development of new cochlear implantation selection criteria.

## 1. Introduction

The great majority of patients with sensorineural hearing loss (SNHL) benefit from conventional hearing aids (HA), but for those with severe-to-profound SNHL for whom HAs are insufficient, cochlear implantation (CI) is currently the standard treatment for rehabilitation. Hearing loss has a wide range of consequences, including impaired verbal communication and social isolation, and has been linked to worsening cognitive functioning and dementia [1]. Patients are also at a higher risk of developing physical and mental illnesses [2]. Improved speech recognition may be achieved with cochlear implants, but they do not restore normal hearing. However, cochlear implantation improves speech understanding [3,4,5,6], can provide spatial hearing and music appreciation, suppresses tinnitus [7], improves quality of life [8,9], and reduces hearing loss-related comorbidities [10]. A meta-analysis by McRackan et al. found that all included studies (*n* = 14) showed significant improvements in both QOL and speech recognition following cochlear implantation [11].

Since the first Danish cochlear implantation in 1982, approximately 4500 patients have been implanted. At the three Danish cochlear implantation centers, nearly 400 implantations are conducted each year [12]. The Danish selection criteria for CI participants were revised in 2014, which builds on criteria described by Dowell et al. [13]. We are currently working to revise the criteria, since CI indications for adult patients have grown considerably since then, making CI an increasingly popular treatment option. It is currently recommended that any adult with a moderate-to-severe sensorineural hearing loss who is unable to comprehend what is said in a quiet conversation without the assistance of mouth reading or in background noise with the assistance of mouth reading be referred for routine assessment for CI candidacy at a CI center. If a patient’s speech recognition score (SRS) is less than 65 percent in a quiet free field setting without the option of lipreading with optimally fitted HAs and/or if the patient’s SRS is less than 45 percent in the CI candidate ear, the patient meets the current Danish national selection criteria for CI. Poor SRS (less than 20%) in a free-field setting with optimally fitted HAs in moderately loud noise (SNR = 0 dB) can also support a candidacy. An otolaryngologist specializing in audiology recommends the patient for implantation when the medical evaluation is completed. Some patients are still eligible even though their speech recognition scores are higher than the above criteria, and they are still considered for CI.

This research aims to provide further evidence on postimplant outcomes using standard speech audiometry and patient-reported outcome measures, allowing evidence-based CI candidate counselling in our department. We are conducting a prospective repeated-measures within-subjects study at our tertiary referral hospital with a cohort of adult CI patients for whom HAs were inadequate to examine the treatment benefits of unilateral CI. Our patient cohort is unbiased and is representative of the patients we encounter at our clinic in terms of etiology, age, and degree of hearing loss. So far, data have been evaluated at 4 months post-implantation, which showed significant improvements in both audiometric and patient-reported outcome measures (PROMs) [5]. Beyond the first post-implantation follow-up, we will investigate whether the speech recognition and PROM outcomes improve, plateau, or deteriorate. We found strong correlations between the PROMs NCIQ and SSQ and a moderate correlation between speech recognition (Dantale) and the PROMs [5]. The current study also aims to find potential correlations between audiometric measures and PROMs at the second post-implantation follow-up.

## 2. Materials and Methods

This study is a prospective longitudinal cohort study with a repeated measures design conducted at our department, the Department of Otorhinolaryngology, Head and Neck Surgery & Audiology, Rigshospitalet. The study included adult patients (aged 18 and above) with SNHL who met the Danish national candidacy criteria [14]. If there were no contraindications, the ear with the worst hearing was selected for implantation. Patients were not included if CI had been performed previously or if the patient could not speak Danish fluently. When CI candidates were offered CI, they were invited to participate in the study. Prior to participation, all patients were informed verbally and in writing and completed informed consent forms. The Danish Data Protection Agency (Reference number: RH-2017-308) and the Danish National Committee on Health Research Ethics (Reference number: H-17034918) approved the study. Between February 2018 and May 2019, patients were enrolled.

### 2.1. Cochlear Implantation

Unilateral cochlear implantation was performed using the round window technique with mastoidectomy and posterior tympanometry. The patient and the speech and language pathologist collaborate to select the implant device brand. However, the electrode type is usually decided upon by the surgeon. All surgery and post-operative procedures were performed by the Department of Otorhinolaryngology, Head and Neck Surgery & Audiology at Rigshospitalet. The patients had three auditory counseling sessions with CI technicians and speech-language pathologists post-operatively. 

### 2.2. Main Outcome Measures

Pure-tone audiometry was performed at baseline (T0) to determine pre-implant hearing capabilities and study eligibility. Patients were also tested at baseline and at two post-implantation follow-ups (T1 and T2) with a test battery including two audiometric tests, Dantale I and Hearing in Noise Test (HINT), as well as two PROMs, a Nijmegen Cochlear Implant Questionnaire (NCIQ), and the 12 items of the Speech, Spatial, and Qualities of Hearing Scale (SSQ-12). The evaluation intervals for T0, T1, and T2 coincided with routine clinical follow-up visits.

### 2.3. Audiometric Test Measures

#### 2.3.1. Pure-Tone Audiometry (PTA) and Speech Recognition Score (SRS)

PTA and SRS were measured at baseline (T0). A pure tone average (PTA6) consisted of six frequencies: 0.25, 0.5, 1, 2, 4, 8 kHz. SRS was measured by the percentage of correctly repeated phonemes from one list of 25 monosyllabic words (Dantale I) containing 80 phonemes. The PTA and SRS were measured in a double-walled, sound-attenuated booth with headsets and without HAs.

#### 2.3.2. Dantale I

Dantale I assesses the speech reception threshold (SRT) measured by means of monosyllabic digit triplets, and a speech recognition score (SRS), measured by the percentage of correctly repeated phonemes from one of eight lists of 25 monosyllabic words containing 80 phonemes [15]. According to ISO 8253-3, the speech recognition material was presented in a quasi-free sound field from a single loudspeaker one meter in front of the patient, while noise was presented from two loudspeakers 45 degrees from the center [16]. Speech was presented at a sound pressure level of 65 dB SPL in quiet and in noise at a signal-to-noise ratio (SNR) of 0 dB. A 1-kHz warble tone was used to calibrate the presentation level. Dantale I is comparable to the consonant-nucleus-consonant (CNC) test used in native English-speaking countries. Data from speech recognition tests in free field in five different listening conditions were included in the study: (1) best aided with possibility of lip-reading, (2) best aided in quiet, (3) best aided in a noise, (4) best aided CI ear only in quiet, (5) best aided CI ear only in noise. Lip movements were visible on a video screen one meter in front of the patient in the audiovisual setting. For unilateral CI testing, contralateral ears were plugged. Preoperative aided tests were with HA to the candidate ear, postoperative tests were with CI. 

#### 2.3.3. Hearing in Noise Test (HINT)

HINT consists of ten lists of twenty sentences each, and it assesses both word and sentence recognition [17]. A Danish version of the HINT was used [18]. The HINT setup was identical to Dantale I. Patients were assessed in four different conditions: best aided in both quiet and noise, with word and sentence scoring for each condition. Speech was delivered at 60 dB SPL in quiet and 65 dB SPL in noise. A SNR of +10 dB was used in the setting with background noise. Thus, HINT testing generated scores for the percentage of correctly perceived words and sentences.

If a patient was unable to respond in Dantale I or HINT owing to hearing loss, or if the patient could not attend the posttest due to COVID-19, the results were declared as missing values (NA).

### 2.4. Patient-Reported Outcome Measures

#### 2.4.1. Nijmegen Cochlear Implant Questionnaire (NCIQ)

The NCIQ is a 60-item questionnaire that assesses hearing ability and quality of life divided in six subdomains: (1) basic sound perception, (2) advanced sound perception, (3) speech production, (4) self-esteem, (5) activity limitations, and (6) social interactions [19]. On a Likert scale, each item may be answered as *never*, *sometimes*, *rarely*, *often*, and *always*, with a corresponding score of 1 to 5, respectively, or with *no relevance*. All subdomains were checked for the number of no relevance-responds, and those with four or more no relevance-responses were omitted. A high NCIQ score is associated with a good self-perceived quality-of-life.

#### 2.4.2. Speech, Spatial, and Qualities of Hearing Scale (SSQ)

The SSQ is a 49-item questionnaire that examines speech perception, sound localization, and sound quality [20]. Noble et al. demonstrated that a simplified version of 12 questions (SSQ12, referred to as SSQ in this study) reflects the original form (SSQ49) and is more practical to use in a clinical context; hence, SSQ12 is used in this study. The SSQ is divided into three subdomains: speech comprehension, spatial hearing, and quality of sound. The SSQ was presented with 10-cm visual analogue scales (VAS). A high SSQ score indicates less limitations in self-reported activity, as well as better speech perception and sound localization.

Patients completed both questionnaires on paper at T0, T1, and T2. If no information was provided in the NCIQ or SSQ, or if the patients did not attend the posttest due to COVID-19, or if the information provided was ambiguous, the scores on the two instruments were declared as missing values (NA).

### 2.5. Statistical Analysis

SPSS [21] was used for statistical analysis, and a GraphPad Prism was used to produce graphs [22]. Demographic data were examined using means, ranges, and percentages. PTA6 and SRS are reported as decibels hearing level (dB HL) and percentage, respectively. Summary audiometric results and patient-reported results were presented as means with 95% confidence intervals and medians with 25 and 75% percentiles at each time point, respectively. Analyses were performed to identify statistically significant differences in the outcome measures between pre-implantation (T0) and post-implantation (T1 and T2). Audiometric measures were analyzed using a repeated measures analysis of variance (ANOVA) to examine changes over time through repeated measures on the same patients. PROMs were analyzed using the Wilcoxon signed-rank test. The Spearman rank correlation analysis, a non-parametric measure of rank correlation (ρ), was used to characterize the relationship between audiometric results and PROMs. All *p* values provided are two-tailed, with a significance level of 0.05.

## 3. Results

### 3.1. Study Subjects

In all, 49 patients were enrolled in the study. Table 1 shows the patient demographics. Due to the circumstances of the COVID-19 pandemic, three patients later withdrew from the study. Another seven patients were excluded: one patient had a bilateral CI performed simultaneously, one patient relocated abroad, one patient was later explanted due to pain and infection around the implanted device, two patients died before the last follow-up, and two patients had comorbidities that excluded them from the study. The remaining 39 patients were between the ages of 28 and 90 at the time of implantation (mean 63), with approximately one-third being female (36%). Twenty-three patients (59%) received implants in their left ear, while 16 (41%) had implants placed in their right ear. Patients reported using HAs on the CI ear for an average of 18 years, ranging from 0 to 57 years, and on the contralateral ear for 19 years (range 0–57 years). A Nucleus CI device was implanted in the majority of patients (Cochlear LTD; *n* = 28; 69%), subdivided between the Nucleus Cochlear CI522 (*n* = 22, 56%) and CI622 (*n* = 6; 13%). Six patients were implanted with Advanced Bionics devices (*n* = 6, 16%), subdivided between ULTRA 3D Midscale (*n* = 3; 8%), 2 (5%) HiRes90K Midscale (*n* = 2, 5%), and HiRes Ultra 3D SlimJ (*n* = 1; 3%). Four patients (10%) received the Oticon Medical Zti EVO, and one (3%) patient received MEDEl Flex 28 Synchrony. Audiometric tests at T1 and T2 were carried out after an average of 235 days and 440 days, respectively. Questionnaires (NCIQ and SSQ) at T1 and T2 were filled out and handed in after an average of 124 days and 426 days, respectively (Table 1).

Table 2 presents individual data on hearing loss etiology, pre-implantation pure-tone audiometry, and speech recognition scores. Fourteen different etiologies were reported, and unknown etiology was most frequently reported (*n* = 14; 36%), followed by late-onset progressive hereditary SNHL (*n* = 9; 23%), hereditary congenital (*n* = 3; 8%), unknown congenital etiology (*n* = 2; 5%), and otosclerosis (*n* = 2; 5%). An additional nine etiologies made up the remaining 27%. Etiology was not considered for further analysis.

### 3.2. Speech Perception Outcomes

#### 3.2.1. Baseline PTA6 and SRS Results

A pre-implant pure-tone audiometry revealed that 24 patients (62%) had moderate to severe SNHL and 15 (38%) had profound SNHL on the CI ear. Thirty-one patients (79%) had moderate to severe SNHL, and 8 (21%) had profound SNHL on the contralateral ear (Table 1 and Table 2). The average pure-tone audiometry for the CI ear was 87 dB HL, whereas the contralateral ear was 80 dB HL. The average speech recognition score (SRS) measured with monosyllable word recognition was 27% (range 0–78) in the CI ears and 41% (range 0–85) in the non-implanted ears. In 22 patients (56%), the SRS was below 65% in the best-aided condition and below 45% in the CI ear. Three patients (8%) had an SRS in best-aided condition higher than 65% and higher than 45% on the CI ear (patient-ID 18, 39, and 42). Bilateral HAs were the best-aided preimplant hearing condition in 33 cases (85%), and unilateral HAs in six cases (15%, three on either side).

Table 3 includes all of the raw data for each subject. While most patients have complete data sets of all their tests, some patients have missing values. Missing values in Dantale I data are due to incomplete testing sequences, meaning that on some occasions the CI ear or contralateral ear was not tested. In the HINT data set, seven patients (18%) had missing tests at all time points. HINT was not performed in these cases because it was too difficult for the patient to complete the test due to poor SRS. Six patients (15%) and eleven patients (28%) had missing values at T2 in the NCIQ and SSQ tests, respectively. Due to COVID-19, several PROM results at T2 were not available during data extraction.

#### 3.2.2. Dantale I Results

**Figure 1:**
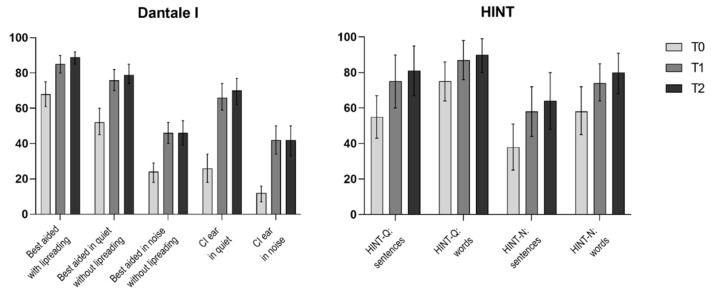
Scores in five Dantale and four HINT conditions at different time points (T0, T1, and T2) of the patient cohort (*n* = 39) are represented in box plots. Columns present means and whiskers present 95% confidence intervals.

In the unaided condition, the Dantale I yielded an average score of 6% (2–10) (all ranges represent the 95 % confidence intervals) in quiet and 3% (1–4) in noise. Table 4 and Figure 1 show the findings of pre- and post-implantation speech recognition tests, along with the *p* value for the overall test of differences between the evaluation time points analyzed with repeated measures ANOVA. For both Dantale I and HINT, the overall test of change revealed a significant improvement in speech recognition in all tested settings (*p* < 0.001). The Bonferroni adjusted pairwise comparisons revealed the highest changes between T0 and T1, but also small insignificant improvements between T1 and T2, indicating that there is an improvement potential beyond T1, even though the rate of progress plateaus at T2.

In the best-aided audiovisual condition with the option of lipreading, patients improved from 68% (62–72) at T0 to 89% (85–92) at T2 (*p* < 0.001), with a 3 percent point rise from T1 to T2. When patients were not allowed to lipread, their performance improved from 52% (45–60) to 79% (74–85) in the quiet condition and from 24% (18–29) to 46% (39–53) in the noise condition. When the CI ear was tested monaurally in both quiet and noise, significant improvements were reported. In quiet, the patients improved from 26% (18–34) at T0 to 70% (62–77) at T2, which was the largest mean difference increase found across all tests, with a 43% (33–53) improvement from T0 to T2 (*p* < 0.001). In noise, patients scored 12% (7–16) at T0 and 42% (33–50) at T2. All Dantale I test conditions showed significant improvements (*p* < 0.001).

#### 3.2.3. HINT Results

The average pre-implantation sentence score for HINT in quiet was 55% (43–67), while the average post-implantation score at T2 was 81 (67–95). The average pre-implantation sentence score for HINT in noise was 38% (25–51) and 64% (48–80) at T2. The average pre-implantation word score for HINT in quiet was 75% (64–86) and the post-implantation score at T2 was 90% (80–99). The average pre-implantation word scores for HINT in noise was 58% (45–72) and the post-implantation score at T2 was 80% (68–91). All HINT test conditions showed significant improvements (*p* < 0.05)

In summary, when pre- and post-implantation tests are compared, patients show significantly better speech perception scores on all audiometric outcome measures (Table 4), with the greatest improvement in the monaural CI ear only test conditions. Between pre-implantation and the first post-implantation follow-up, the greatest significant change in speech perception scores occurred. 

### 3.3. Patient-Reported Outcomes

**Figure 2:**
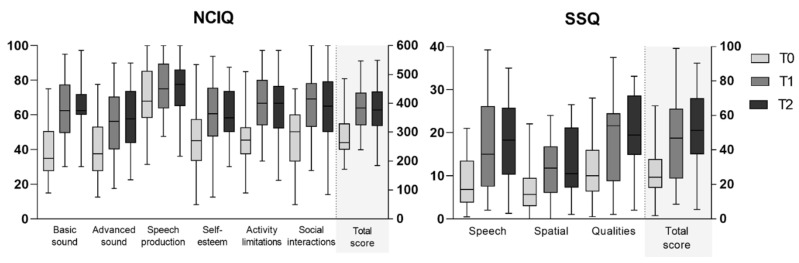
Subdomain percentile scores for the Nijmegen Cochlear Implant Questionnaire (NCIQ) and the Speech, Spatial, and Qualities of Hearing Scale (SSQ) at T0, T1 and T2. Boxes present the median and the 25th and 75th percentiles. Whiskers present the maximum and minimum values. Total scores are presented on the right y axis.

Table 5 and Figure 2 present PROM results from pre-implantation to post-implantation.

#### 3.3.1. NCIQ Results

The median NCIQ total score improved by 113 points (264 (239–330) at T0 vs. 377 (321–441) at T2, *p* < 0.001). All NCIQ subdomain scores improved significantly from T0 to T2 (*p* < 0.05) (Table 5 and Figure 2). The greatest significant improvements were seen in the basic and advanced sound perception subdomains, whereas the least improvement was seen in the speech production subdomain. These findings suggest that after being implanted, patients had enhanced basic and advanced sound perception, less limitations in activity level and social interaction, as well as higher self-esteem. This also strongly suggests that patients’ general quality of life improves after being implanted. 

#### 3.3.2. SSQ12 Results

The SSQ total score increased with 27 points (24 (18–33) at T0 vs. 51 (37–70) at T2, *p* < 0.001). The scores on all SSQ subdomains improved significantly (Table 5 and Figure 2).

While there is a significant increase in NCIQ and SSQ scores between baseline and post-implantation, there is only little improvement between 4 and 14 months after implantation. After the first post-implantation follow-up, both audiometric and patient-reported outcome measures, as assessed by NCIQ and SSQ, begin to plateau.

### 3.4. Comparisons between Audiometric and Patient-Reported Outcome Measures

At the second post-implantation follow-up (T2), the four Dantale conditions (without the best-aided audiovisual condition), have significant positive correlations with the NCIQ total score (ρ = 0.44–0.53, Table 6). The SSQ total score and Dantale I scores showed significant correlations (range, ρ = 0.30–0.44), however best-aided in noise without lipreading and the CI ear in noise conditions were insignificant (*p =* 0.124 and *p =* 0.102, respectively). Dantale I and NCIQ revealed a statistically significant correlation at the 14-month evaluation, meaning that patients who do well on Dantale also perform well on NCIQ and, to some extent, on SSQ, and vice versa. HINT results do not correlate with NCIQ and SSQ results (*p* > 0.05).

## 4. Discussion

In this prospective follow-up study, we combined audiometric test results (Dantale and HINT) and PROMs (NCIQ and SSQ) to investigate whether statistically significant changes had occurred over time and if there was a correlation between the measures. In the previous study, patients showed excellent improvement in Dantale and moderately to excellent improvements in HINT, and the NCIQ showed a moderate positive correlation with audiometric test scores. When compared to HA in the best-aided condition prior to implantation, speech recognition scores assessed with Dantale I and HINT in quiet and noise were significantly improved at 4 and 14 months following implantation. The greatest improvements occurred before T1, albeit non-significant minor improvements were reported at T2, and we observed considerable individual variation in the test results.

Speech perception improved significantly between the baseline and the 14-month follow-up, with excellent improvements in the CI-monaurally-aided condition in both quiet and noise. On average, Dantale I scores improved by 29 percentage points, and HINT scores improved by 22 percentage points. Our results agree with previous studies in the field [3,23,24,25]. Sladen et al., Kelsall et al., and Buchman et al. reported average improvements in speech recognition on the CNC word test of 57 percent, 51 percent, and 41 percent, respectively [3,6,25], at 12 months post-implantation. Firszt et al. found that half of the individuals scored 80 percent or better on the HINT sentences in a quiet setting. Several other research studies have found comparable improvements in audiometric outcomes over time [23,26,27,28,29,30,31], with most of the improvements happing before 6 months of CI usage. Cusumano et al. reported that word scores continued to improve 3 years after implantation, but at a slower rate [23].

In addition to improved speech recognition, the present study also demonstrates significant improvements in quality of life measured by NCIQ and SSQ total scores as well as subdomain scores. The NCIQ total score improved by 43 percent from 264 (239–330) to 377 (321–441), whereas the SSQ total score improved by 113 percent from 24 (18–35) to 51 (37–70). All NCIQ subdomains, including basic sound perception, advanced sound perception, speech production, self-esteem, activity, and social interactions also improved significantly between the baseline and 14 months of follow-up. The same is observed for the SSQ: The three subdomains (speech, spatial, and qualities) all improved significantly. Both PROMs are frequently utilized in other studies, with the NCIQ being the more common, and several studies have reported similar results on both the NCIQ [8,23,26,28,31,32,33,34,35,36] and the SSQ [27,30,37,38,39].

Boisvert et al. discovered 16 different PROMs being used to evaluate the benefits of CI. The NCIQ and SSQ were the most utilized across 52 research studies, with 17 percent using the NCIQ and 15 percent using the SSQ. Our findings are consistent with those of other studies that found a significant correlation between NCIQ scores and speech perception test scores [26,28,32,36]. A systematic review and a scoping review both reveal moderate correlations between PROM outcomes and audiometric outcomes [4,40]. In recent studies, Vasil et al., Plath et al., and Sladen et al. show moderate correlations between the NCIQ subdomains and audiometric measures, and the physical subdomain, especially, stands out, comprising basic and advanced speech perception and speech production [8,25,34]. This finding illustrates that CI patients have increased their capacity for audiovisual speech perception, which is not completely assessed in clinical audiometric measures. McRackan et al. showed in a systematic review that the hearing specific subdomains (basic and advanced hearing) were the primary drivers of quality of life improvement and less so for the other four subdomains (speech production, self-esteem, activity limitation, and social interaction). They argue that CI has a stronger effect on sound processing than non-hearing subdomains, and that non-hearing subdomains simply do not represent what CI patients perceive to be crucial for improving their quality of life [11]. The NCIQ may correlate well with audiometric measures because the questionnaire is designed particularly for CI patients. Some studies also find no correlation with audiometric measures. Olze et al., who evaluated CI benefits in elderly patients, found no correlation between the results of the monosyllable speech perception test and the NCIQ scores after cochlear implantation [41], which could be attributed to the small number of patients (*n* = 39) included in the study combined with subgroup analyses of hearing loss severity. Vasil et al. showed a correlation at the subdomain level, but the total NCIQ was not strongly correlated with any audiometric outcome measures [8].

Other research studies have revealed moderate correlations between SSQ scores and audiometric measures, and our findings are consistent with these [38,39,42]. Lenarz et al. found that the mean SSQ overall score and mean subdomain scores both improved significantly. Mean scores were constant for over a year after implantation, despite significant individual variability [38]. According to Gatehouse and Noble (10), the SSQ was created to evaluate a range of hearing disabilities divided into three domains and is therefore an instrument for evaluating hearing rehabilitation therapies such as CI, but also conventional hearing aids and bone anchored hearing systems. Although validation studies on the shorter 12-question version of the SSQ have been conducted, this may lead to oversimplification, rendering the instrument incapable of assessing and evaluating the complexity of everyday hearing conditions. For instance, the spatial hearing subdomain contains only three questions reflecting aspects of the temporal and spatial dynamics of hearing.

In terms of the correlation between speech perception and PROMS, the current study adds to the growing body of data indicating that the effects of cochlear implantation go beyond enhanced speech perception. Patients who acquire modest levels of speech perception report considerable improvements in their quality of life.

### Study Strengths and Limitations

The inclusion of a variety of audiometric and patient-reported outcome measures to characterize the benefit of cochlear implantation in adults is one of the study’s strengths. When evaluating speech perception, noise conditions give a more accurate representation of real-life hearing conditions. Omitting noise conditions could lead to an overestimation of benefits from CI. We examined the outcomes at baseline and two times after implantation in a repeated measures research design, increasing the validity of the data. Hence, the progression of treatment benefits may be studied in more depth. Another study strength is that the study includes the two most widely used PROMs. As mentioned by McRackan et al. [33], there is a need for a more regular use of PROMs specific to CI, and by conducting correlation analyses we can improve these tools used by clinicians.

This study has a number of limitations. Although the sample size was adequate for our study’s purpose of assessing outcomes after cochlear implantation, it was too small for us to perform multi-factor analyses. Secondly, at our department we use Dantale I with phoneme scoring, but in the majority of studies word scoring has been applied. Gifford et al. found a reverse relationship between CNC phoneme scoring and HINT scores, leading to higher CNC scores compared to HINT scores [43]. As a result, phoneme scoring makes it difficult to compare the findings to those of other studies using CNC word tests, and it may influence how the results are understood, but it does not invalidate the results. Other limitations of the present studies include the fact that the research cohort is comprised of a heterogeneous group of patients in terms of age, hearing loss etiology, and implant device, and as our results indicate, there is considerable individual variability among the study subjects. The almost overlapping test intervals in time between each follow-up examination in the clinic may potentially contribute to this variability. This can contribute to non-significant differences between T1 and T2. Another limitation of the present study is the missing data, especially on the follow-up and mostly seen in HINT and SSQ results. Some HINT data were missing either because they were omitted owing to poor Dantale results, poor cooperation from elderly patients who struggled with fatigue or attention throughout the examination, or department lockdown during the COVID-19 pandemics, to name a few reasons. HINT results may then be overestimated as poorer performers are excluded. Some SSQ data were lost to follow-up. We did not examine the possible impact of reduced cognitive capacity and comorbidities, nor did we include data on cigarette and alcohol consumption, as well as physical activity. A better understanding of these aspects in future studies may allow us to distinguish between low-performing and high-performing patients.

## 5. Conclusions

The majority of patients in our study benefited from CI in terms of speech perception and quality of life, and they experienced considerable improvements during the first postoperative follow-up, which remained stable and plateaued at the second follow-up. In this longitudinal study of a cohort of adult SNHL patients treated with unilateral CI, Dantale I and HINT audiometric tests, as well as patient-reported outcome measures including the NCIQ and SSQ, revealed significant improvements in speech perception and quality of life. In most conditions, NCIQ and SSQ showed a moderate correlation with Dantale I. HINT showed no correlation with NCIQ or SSQ 14 months after implantation. The use of audiometric and CI-specific patient-reported outcome measures in our study provides detailed evidence of the treatment benefits of cochlear implantation in adults, which may help physicians to advise patients in their treatment decision and align treatment benefit expectations. This study, along with other recent studies on the benefits of CI on hearing, is helping to establish the framework for the development of new Danish CI selection criteria.

## Figures and Tables

**Table 1:** Demographic characteristics of the 39 study patients.

Age at Implantation	28–90 Years (Mean 64)
Gender	15 females (38%), 24 males (62%)
Implanted side	23 left (59%), 16 right (41%)
Duration of hearing aids	
CI ear	18 years (range 0–57 years)
Contralateral ear	19 years (range 0–57 years)
Hearing loss degree	
CI ear	
Moderate–severe (56–90 dB)	24 (62%)
Profound (91+ dB)	15 (38%)
Contralateral ear	
Moderate–severe (56–90 dB)	31 (79%)
Profound (91+ dB)	8 (21%)
Type of implant	1 (3%) Advanced Bionics HiRes Ultra 3D SlimJ
	2 (5%) Advanced Bionics HiRes90K Midscale
	3 (8%) Advanced Bionics ULTRA 3D Midscale
	1 (3%) MED-EL Flex 28 Synchrony
	22 (56%) Nucleus Cochlear CI522
	6 (13%) Nucleus Cochlear CI622
	4 (10%) Oticon Medical Zti EVO
Audiometric test—days after implantation	
T1	235 days, 110–330 (mean, range) ~ 8 months
T2	440 days, 343–636 (mean, range) ~ 15 months
PROMs—days after implantation	
T1	124 days, 66–241 (mean, range) ~ 4 months
T2	426 days, 301–569 (mean, range) ~ 14 months

**Table 2:** The baseline info on hearing loss etiology and pre-operative hearing for the 39 included patients.

ID	Etiology	Gender	Age	PTA6 Operated Ear	PTA6 Contralateral Ear	SRS CI Ear	SRS Contralateral Ear
			(Years)	(dB HL)	(dB HL)	(%)	(%)
1	Ménière’s disease	M	77	77	52	45	80
2	Otitis media	M	73	110	106	9	53
3	Otosclerosis	F	53	120	78	0	68
4	Congenital (unknown etiology)	F	46	108	115	0	0
5	Unknown	M	70	98	70	0	28
6	Unknown	M	74	98	86	48	33
7	Late-onset progressive hereditary	F	69	77	83	60	60
8	Superficial Siderosis	F	67	68	59	24	24
9	Late-onset progressive hereditary	M	69	87	89	24	32
10	Hereditary congenital	M	69	111	90	0	45
11	Unknown	M	81	78	69	14	56
12	Unknown	M	70	108	78	0	85
13	Usher syndrome	F	60	92	86	50	68
14	Late-onset progressive hereditary	F	62	76	71	29	28
15	Late-onset progressive hereditary	M	60	75	78	45	55
16	Unknown	F	75	103	82	0	9
17	Late-onset progressive hereditary	M	59	108	108	0	0
18	Unknown	M	88	67	64	56	49
19	Ototoxicity	F	90	79	77	0	0
20	Unknown	M	74	75	63	14	21
21	Late-onset progressive hereditary	M	80	63	55	20	35
22	Unknown	M	73	78	77	39	28
23	Unknown	M	56	99	74	0	77
24	Neurofibromatosis	M	56	68	72	15	35
25	Congenital (unknown etiology)	M	53	103	84	35	85
26	Vestibular schwannoma	M	75	83	118	50	0
27	Pendred syndrome	F	28	84	70	33	70
28	Hereditary congenital	F	43	96	87	48	60
29	Unknown	F	40	86	118	40	0
30	Late-onset progressive hereditary	M	72	78	74	35	65
31	Unknown	F	85	115	82	0	10
32	Unknown	F	30	88	63	31	68
33	Otosclerosis	M	81	88	86	20	30
34	Pneumococcal meningitis	M	69	56	53	30	40
35	Late-onset progressive hereditary	M	87	79	59	55	40
39	Unknown	F	38	73	118	75	0
40	Unknown	M	79	73	67	41	68
42	Late-onset progressive hereditary	F	36	73	68	78	72
43	Hereditary congenital	M	33	104	91	8	40

F: female; M: male; PTA6: pure-tone average of frequencies 0.25, 0.5, 1, 2, 4, 8 kHz (headphone based); SRS, speech recognition score (in a quiet setting at individually determined most comfortable loudness levels using headphones).

**Table 3:** Individual patient outcomes for audiometric test measures (Dantale I and HINT) and patient-reported outcome measures (NCIQ and SSQ) at T0/T1/T2.

ID	Dantale I					HINT				NCIQ	SSQ
	Best Aided with Lipreading	Best Aided in Quiet without Lipreading	Best Aided in Noise without Lipreading	CI Ear in Quiet	CI Ear in Noise	HINT-Q: Sentences	HINT-Q: Words	HINT-N: Sentences	HINT-N: Words		
	(%)	(%)	(%)	(%)	(%)	(%)	(%)	(%)	(%)	Total Score	Total Score
1	85/94/89	85/89/84	51/43/60	46/53/51	29/21/38	50/75/100	80/92/100	35/68/80	62/79/88	257/293/265	NA/42/42
2	86/98/90	65/81/88	28/49/54	NA/81/76	NA/45/54	45/85/90	69/96/98	15/45/70	18/67/78	232/268/273	30/37/32
3	59/94/91	34/91/96	21/53/59	NA/71/76	NA/44/59	NA/95/100	NA/98/100	NA/60/80	NA/83/90	239/289/377	27/34/NA
4	40/63/86	2/44/59	NA/NA/NA	2/44/59	NA/NA/NA	NA/NA/NA	NA/NA/NA	NA/NA/NA	NA/NA/NA	329/323/348	25/16/47
5	41/94/99	31/88/96	NA/49/55	9/71/88	NA/49/55	NA/95/80	NA/98/91	NA/65/90	NA/83/96	246/448/487	26/77/84
6	86/86/84	49/75/78	NA/19/33	8/48/58	NA/19/33	10/NA/65	28/NA/87	NA/NA/20	8/NA/44	263/425/410	23/50/56
7	NA/98/96	45/93/83	40/71/74	45/91/83	36/65/74	70/95/90	86/99/98	35/75/80	63/91/93	377/423/435	64/57/62
8	88/79/84	78/46/65	30/30/21	48/46/65	NA/30/21	35/20/20	46/33/33	15/NA/NA	33/NA/NA	171/248/185	5/20/5
9	70/86/94	30/76/78	10/36/14	16/73/78	4/36/14	NA/60/60	NA/78/80	NA/25/40	NA/56/65	253/418/403	39/65/59
10	62/93/90	34/68/75	3/58/53	NA/68/75	NA/58/53	NA/20/40	14/42/68	NA/10/20	NA/34/51	486/533/533	54/90/74
11	84/99/99	75/91/95	64/54/54	29/74/90	26/40/53	NA/NA/NA	NA/NA/NA	NA/NA/NA	NA/NA/NA	266/412/328	27/56/42
12	89/98/96	77/88/89	36/58/55	35/88/86	NA/56/55	70/100/100	92/100/100	60/75/80	81/91/92	234/328/315	18/31/41
13	86/94/95	63/91/88	24/36/39	18/74/79	18/36/30	55/100/100	80/100/100	55/75/85	73/84/96	373/487/NA	46/72/74
14	66/88/98	70/93/91	27/60/63	70/86/81	27/60/55	60/90/95	80/96/99	30/60/65	61/76/84	293/434/447	16/57/37
15	92/100/99	69/83/89	21/64/69	36/83/89	13/64/69	75/100/85	88/100/95	45/70/60	65/82/78	240/385/425	20/58/90
16	38/53/61	8/40/54	NA/5/4	1/28/26	NA/5/NA	NA/NA/5	NA/NA/22	NA/NA/NA	NA/NA/NA	NA/226/NA	NA/NA/NA
17	58/NA/63	28/NA/21	NA/NA/1	10/NA/15	NA/NA/1	NA/NA/NA	5/NA/NA	NA/NA/NA	NA/NA/NA	261/341/NA	29/9/NA
18	83/88/NA	66/85/85	14/44/51	40/69/73	6/33/41	NA/NA/NA	NA/NA/NA	NA/NA/NA	NA/NA/NA	373/508/549	32/71/71
19	26/76/84	7/73/76	NA/NA/11	NA/65/76	NA/NA/NA	NA/NA/NA	NA/NA/NA	NA/NA/NA	NA/NA/NA	256/341/364	17/45/NA
20	57/88/86	39/66/74	15/30/30	9/46/45	4/19/28	NA/NA/NA	NA/NA/NA	NA/NA/NA	NA/NA/NA	311/355/360	16/25/NA
21	94/96/91	70/84/80	21/43/45	31/84/68	3/43/33	95/90/85	99/98/93	80/85/75	91/93/88	325/396/368	52/56/72
22	79/96/95	65/98/94	13/73/69	23/98/88	13/73/61	40/85/NA	74/96/NA	40/NA/NA	64/NA/NA	444/547/532	66/64/NA
23	89/98/98	61/96/93	31/49/68	NA/78/90	NA/49/59	85/100/NA	95/100/NA	35/90/NA	61/98/NA	238/308/NA	19/16/NA
24	65/88/85	59/81/78	14/38/36	NA/81/78	NA/38/36	NA/84/75	NA/94/93	NA/49/65	NA/65/87	259/371/404	9/19/NA
25	87/100/96	70/81/91	44/60/54	NA/61/73	NA/31/26	35/70/85	59/86/93	40/40/15	56/66/44	310/414/433	38/49/69
26	40/75/86	29/84/84	8/46/55	29/84/84	8/46/55	NA/70/75	NA/85/90	NA/20/50	NA/42/72	340/339/374	32/16/40
27	88/93/91	74/89/95	40/75/45	41/68/69	11/33/44	50/65/80	72/87/89	15/45/60	42/78/78	384/461/478	47/99/NA
28	88/96/99	79/98/91	20/66/73	33/91/88	16/66/73	75/80/100	85/95/100	20/93/90	47/80/92	332/526/529	18/74/60
29	39/83/93	34/54/69	16/30/51	NA/54/69	NA/30/51	NA/5/15	NA/35/54	NA/NA/NA	NA/NA/21	177/239/291	9/NA/19
30	73/NA/96	64/91/93	24/85/63	41/85/89	4/85/63	80/85/100	90/99/100	75/80/90	80/94/98	340/487/432	24/73/51
31	20/48/69	6/23/33	NA/NA/NA	NA/23/33	NA/NA/NA	NA/NA/NA	NA/NA/NA	NA/NA/NA	NA/NA/NA	315/384/332	24/19/22
32	70/93/98	49/75/90	46/28/49	NA/75/85	NA/28/49	30/95/95	74/95/99	15/45/90	63/66/98	206/385/474	18/35/66
33	85/96/90	58/74/73	29/43/41	NA/65/73	NA/43/30	NA/78/85	NA/87/90	NA/60/35	NA/88/61	194/495/421	7/86/52
34	52/53/64	50/40/30	45/45/24	25/40/30	16/45/24	NA/NA/NA	9/15/NA	NA/NA/NA	NA/NA/NA	288/287/304	29/34/26
35	43/65/69	23/48/41	10/18/1	10/44/31	8/18/NA	NA/NA/NA	NA/NA/NA	NA/NA/NA	NA/NA/NA	235/308/290	19/31/18
39	74/79/90	74/79/81	44/45/39	74/79/81	44/45/39	55/25/30	80/71/67	55/5/NA	73/35/35	210/355/351	21/51/76
40	70/58/80	56/53/69	17/30/16	19/43/64	15/30/14	40/75/NA	65/86/NA	25/35/NA	50/65/NA	242/329/308	20/15/40
42	85/98/100	72/94/95	42/70/74	57/86/86	36/70/68	35/NA/NA	65/NA/NA	20/NA/NA	54/NA/NA	322/NA/NA	40/50/NA
43	58/86/85	59/88/91	10/53/69	19/63/73	NA/46/55	10/NA/NA	41/NA/NA	NA/NA/NA	16/NA/NA	328/418/NA	2/41/NA

Test scores for all patients enrolled presented as T0/T1/T2; HINT: Hearing in Noise Test; NCIQ: Nijmegen Cochlear Implant Questionnaire; SSQ: Speech, Spatial, and Qualities of Hearing Scale; NA: Not available.

**Table 4:** Summary of audiometric data showing significant improvements between the evaluation time points T0 (baseline), T1, and T2 (post-implantation) using a repeated measures ANOVA. Mean difference between T0 and T2, as well as *p* values end effect sizes ($\eta_{partial}^{2}$) are reported.

		*n*	T0	T1	T2	Mean Difference (T0–T2) ^a^	*p*-Value	$\eta_{partial}^{2}$
Dantale								
Best aided with lipreading	(%)	35	68 (61–75)	85 (80–90)	89 (85–92)	21 (13–28)	<0.001	0.588
Best aided in quiet without lipreading	(%)	38	52 (45–60)	76 (70–82)	79 (74–85)	27 (19–35)	<0.001	0.667
Best aided in noise without lipreading	(%)	36	24 (18–29)	46 (40–52)	46 (39–53)	22 (13–31)	<0.001	0.516
CI ear in quiet	(%)	31	26 (18–34)	66 (59–74)	70 (62–77)	43 (33–53)	<0.001	0.794
CI ear in noise	(%)	31	12 (7–16)	42 (34–50)	42 (33–50)	30 (20–40)	<0.001	0.663
HINT								
HINT-Q: sentences	(%)	16	55 (43–67)	75 (60–90)	81 (67–95)	26 (9–43)	<0.001	0.528
HINT-Q: words	(%)	16	75 (64–86)	87 (76–98)	90 (80–99)	15 (3–26)	0.003	0.446
HINT-N: sentences	(%)	15	38 (25–51)	58 (44–72)	64 (48–80)	26 (1–50)	0.012	0.370
HINT-N: words	(%)	15	58 (45–72)	74 (64–85)	80 (68–91)	21 (4–39)	0.006	0.432

Test scores are presented as means and 95% confidence interval in parantheses; CI: cochlear implant; HINT: Hearing in Noise test. ^a^ Bonferroni adjusted pairwise comparison between T0 and T2.

**Table 5:** Summary of patient-reported outcome measures showing significant improvements between the evaluation time points T0 (baseline), T1, and T2 (post-implantation) using a Wilcoxon signed rank test. Z-statistics and *p* values are reported.

	T0	T1	T2	Wilcoxon Signed Rank Test			
				T0–T1		T0–T2	
				Z	*p*-Value	Z	*p*-Value
NCIQ total score	264 (239–330)	384 (324–437)	377 (321–441)	−5.2	<0.001	−5.0	<0.001
Physical subdomains							
Basic sound perception	35 (28–51)	63 (49–78)	63 (60–72)	−5.2	<0.001	−4.9	<0.001
Advanced sound perception	38 (28–53)	56 (40–71)	58 (44–74)	−4.8	<0.001	−4.4	<0.001
Speech production	68 (58–85)	75 (64–90)	78 (65–86)	−2.6	0.009	−2.1	0.024
Psychological subdomain							
Self-esteem	45 (33–58)	61 (48–76)	58 (50–74)	−4.5	<0.001	−4.1	<0.001
Social subdomains							
Activity limitations	45 (37–53)	67 (54–80)	67 (52–77)	−5.2	<0.001	−4.8	<0.001
Social interactions	50 (33–60)	69 (53–78)	65 (50–79)	−4.8	<0.001	−4.7	<0.001
SSQ total score	24 (18–35)	47 (23–64)	51 (37–70)	−3.7	<0.001	−4.1	<0.001
Speech	7 (4–14)	15 (8–26)	18 (10–26)	−4.4	<0.001	−4.4	<0.001
Spatial	6 (3–10)	12 (6–17)	11 (7–21)	−3.6	<0.001	−3.2	0.002
Qualities	10 (6–16)	22 (9–25)	20 (15–29)	−2.9	0.004	−4.0	<0.001

Test scores are presented as medians and 25th and 75th percentiles in parentheses; NCIQ, Nijmegen Cochlear Implant Questionnaire; SSQ, Speech, Spatial, and Qualities of Hearing Scale.

**Table 6:** Correlations between audiometric and patient-reported outcome measures at the second post-implantation follow-up (T2) using Spearman coefficients.

	Dantale				HINT			
Test Condition	Best Aided in Quiet without Lipreading	Best Aided in Noise without Lipreading	CI Ear in Quiet	CI Ear in Noise	HINT-Q: Sentences	HINT-Q: Words	HINT-N: Sentences	HINT-N: Words
NCIQ total score	0.53 ***	0.44 **	0.48 **	0.47 **	0.15	0.13	0.21	0.26
SSQ total score	0.45 *	0.30	0.45 *	0.32	−0.02	0.02	0.07	0.12

CI: cochlear implant; HINT-Q/N: Hearing in noise test quiet/noise; NCIQ: Nijmegen cochlear implant questionnaire; SSQ: Speech, spatial, and qualities of hearing. * *p* < 0.05; ** *p* < 0.01; *** *p* < 0.001.

## Data Availability

The raw data supporting the conclusions of this article will be made available by the authors, without undue reservation.

## Abbreviations

CI	cochlear implant
dB	decibel
HA	hearing aid
HINT	hearing in noise test
NCIQ	Nijmegen Cochlear Implant Questionnaire
PROM	patient-reported outcome measure
PTA6	pure-tone audiometry average for six frequencies
SNHL	sensorineural hearing loss
SNR	signal-to-noise ratio
SRS	speech recognition score
SSQ	Speech, Spatial, and Qualities of Hearing Scale

## References

[1] Choi J.S., Contrera K.J., Betz J.F., Blake C.R., Niparko J.K., Lin F.R. (2014). Long-Term Use of Cochlear Implants in Older Adults. Otol. Neurotol..

[2] Contrera K.J., Sung Y.K., Betz J., Li L., Lin F.R. (2017). Change in loneliness after intervention with cochlear implants or hearing aids. Laryngoscope.

[3] Kelsall D., Lupo J., Biever A. (2021). Longitudinal outcomes of cochlear implantation and bimodal hearing in a large group of adults: A multicenter clinical study. Am. J. Otolaryngol..

[4] Gaylor J.M., Raman G., Chung M., Lee J., Rao M., Lau J., Poe D.S. (2013). Cochlear Implantation in Adults: A Systematic Review and Meta-analysis. JAMA Otolaryngol. Head Neck Surg..

[5] West N.C., Kressner A.A., Baungaard L.H., Sandvej M.G., Bille M., Cayé-Thomasen P. (2020). Nordic results of cochlear implantation in adults: Speech perception and patient reported outcomes. Acta Oto-Laryngol..

[6] Buchman C.A., Herzog J.A., McJunkin J.L., Wick C.C., Durakovic N., Firszt J.B., Kallogjeri D. (2020). Assessment of Speech Understanding After Cochlear Implantation in Adult Hearing Aid Users. JAMA Otolaryngol. Head Neck Surg..

[7] Yuen E., Ma C., Nguyen S.A., Meyer T.A., Lambert P.R. (2021). The Effect of Cochlear Implantation on Tinnitus and Quality of Life: A Systematic Review and Meta-analysis. Otol. Neurotol..

[8] Vasil K.J., Lewis J., Tamati T., Ray C., Moberly A.C. (2020). How Does Quality of Life Relate to Auditory Abilities? A Subitem Analysis of the Nijmegen Cochlear Implant Questionnaire. J. Am. Acad. Audiol..

[9] Olze H., Szczepek A.J., Haupt H., Zirke N., Graebel S., Mazurek B. (2012). The impact of cochlear implantation on tinnitus, stress and quality of life in postlingually deafened patients. Audiol. Neurootol..

[10] Dammeyer J., Chapman M. (2017). Prevalence and characteristics of self-reported physical and mental disorders among adults with hearing loss in Denmark: A national survey. Soc. Psychiatry Psychiatr. Epidemiol..

[11] McRackan T.R., Bauschard M., Hatch J.L., Franko-Tobin E., Droghini H.R., Velozo C.A., Nguyen S.A., Dubno J.R. (2018). Meta-analysis of Cochlear Implantation Outcomes Evaluated With General Health-related Patient-reported Outcome Measures. Otol. Neurotol..

[12] Sundhedsdatastyrelsen (2020). Cochlear Implant in Denmark 2005–2018.

[13] Dowell R.C., Hollow R., Winton E. (2004). Outcomes for Cochlear Implant Users With Significant Residual Hearing: Implications for Selection Criteria in Children. Arch. Otolaryngol. Head Neck Surg..

[14] Danish Society of Otorhinolaryngology Head and Neck Surgery (2014). Udredning af voksne til CI. http://dsohh.dk/wp-content/uploads/2015/04/DSOHH-KKR-CI-voksne1.pdf.

[15] Elberling C., Ludvigsen C., Lyregaard P.E. (1989). Dantale: A new danish speech material. Scand. Audiol..

[16] FORCE Technology, Teknisk Audiologisk Laboratorium (2020). Instructions for Setting Up and Calibrating Equipment for Audiometry in Free Field Translated from Danish. FORCE Technology.

[17] Nilsson M., Soli S.D., Sullivan J.A. (1994). Development of the Hearing In Noise Test for the measurement of speech reception thresholds in quiet and in noise. J. Acoust. Soc. Am..

[18] Nielsen J.B., Dau T. (2011). The Danish hearing in noise test. Int. J. Audiol..

[19] Hinderink J.B., Krabbe P.F.M., Van Den Broek P. (2000). Development and application of a health-related quality-of-life instrument for adults with cochlear implants: The Nijmegen Cochlear Implant Questionnaire. Otolaryngol. Head Neck Surg..

[20] Gatehouse S., Noble W. (2004). The Speech, Spatial and Qualities of Hearing Scale (SSQ). Int. J. Audiol..

[21] IBM Corp (2017). IBM SPSS Statistics for Windows.

[22] Graphpad Prism Softare (2020). Graphpad Prism, Version 9.0.0 for Windows.

[23] Cusumano C., Friedmann D.R., Fang Y., Wang B., Roland J.T.J., Waltzman S.B. (2017). Performance Plateau in Prelingually and Postlingually Deafened Adult Cochlear Implant Recipients. Otol. Neurotol..

[24] Budenz C.L., Cosetti M.K., Coelho D.H., Birenbaum B., Babb J., Waltzman S., Roehm P.C. (2011). The Effects of Cochlear Implantation on Speech Perception in Older Adults. J. Am. Geriatr. Soc..

[25] Sladen D.P., Peterson A., Schmitt M., Olund A., Teece K., Dowling B., DeJong M., Breneman A., Beatty C.W., Carlson M.L. (2017). Health-related quality of life outcomes following adult cochlear implantation: A prospective cohort study. Cochlear Implant. Int..

[26] Häußler S.M., Knopke S., Wiltner P., Ketterer M., Grabel S., Olze H. (2019). Long-term Benefit of Unilateral Cochlear Implantation on Quality of Life and Speech Perception in Bilaterally Deafened Patients. Otol. Neurotol..

[27] Wallhäusser-Franke E., Balkenhol T., Hetjens S., Rotter N., Servais J.J. (2018). Patient Benefit Following Bimodal CI-provision: Self-reported Abilities vs. Hearing Status. Front. Neurol..

[28] Hirschfelder A., Gräbel S., Olze H. (2008). The impact of cochlear implantation on quality of life: The role of audiologic performance and variables. Otolaryngol. Head Neck Surg..

[29] Olze H., Gräbel S., Förster U., Zirke N., Huhnd L.E., Haupt H., Mazurek B. (2012). Elderly patients benefit from cochlear implantation regarding auditory rehabilitation, quality of life, tinnitus, and stress. Laryngoscope.

[30] Tavora-Vieira D., Marino R., Acharya A., Rajan G.P. (2015). The impact of cochlear implantation on speech understanding, subjective hearing performance, and tinnitus perception in patients with unilateral severe to profound hearing loss. Otol. Neurotol..

[31] Carlson M.L., Driscoll C.L.W., Gifford R.H., McMenomey S.O. (2012). Cochlear implantation: Current and future device options. Otolaryngol. Clin. N. Am..

[32] Cohen S.M., Labadie R.F., Dietrich M.S., Haynes D.S. (2004). Quality of Life in Hearing-Impaired Adults: The Role of Cochlear Implants and Hearing Aids. Otolaryngol. Head Neck Surg..

[33] McRackan T.R., Bauschard M., Hatch J.L., Franko-Tobin E., Droghini H.R., Nguyen S.A., Dubno J.R. (2018). Meta-analysis of quality-of-life improvement after cochlear implantation and associations with speech recognition abilities. Laryngoscope.

[34] Plath M., Marienfeld T., Sand M., van de Weyer P.S., Praetorius M., Plinkert P.K., Baumann I., Zaoui K. (2021). Prospective study on health-related quality of life in patients before and after cochlear implantation. Eur. Arch. Oto-Rhino-Laryngol..

[35] Hänsel T., Gauger U., Bernhard N., Behzadi N., Ventura M.E.R., Hofmann V., Olze H., Knopke S., Todt I., Coordes A. (2018). Meta-analysis of subjective complaints of vertigo and vestibular tests after cochlear implantation. Laryngoscope.

[36] Damen G.W.J.A., Beynon A.J., Krabbe P.F.M., Mulder J.J.S., Mylanus E.A.M. (2007). Cochlear implantation and quality of life in postlingually deaf adults: Long-term follow-up. Otolaryngol. Head Neck Surg..

[37] Fuller C., Free R., Maat B., Başkent D. (2012). Musical background not associated with self-perceived hearing performance or speech perception in postlingual cochlear-implant users. J. Acoust. Soc. Am..

[38] Lenarz T., Muller L., Czerniejewska-Wolska H., Varela H.V., Dotú C.O., Durko M., Irujo A.H., Piszczatowski B., Zadrożniak M., Irwin C. (2017). Patient-Related Benefits for Adults with Cochlear Implantation: A Multicultural Longitudinal Observational Study. Audiol. Neurootol..

[39] Dingemanse G., Goedegebure A. (2020). The relation of hearing-specific patient-reported outcome measures with speech perception measures and acceptable noise levels in cochlear implant users. Int. J. Audiol..

[40] Boisvert I., Reis M., Au A., Cowan R., Dowell R.C. (2020). Cochlear implantation outcomes in adults: A scoping review. PLoS ONE..

[41] Olze H., Szczepek A.J., Haupt H., Förster U., Zirke N., Gräbel S., Mazurek B. (2011). Cochlear implantation has a positive influence on quality of life, tinnitus, and psychological comorbidity. Laryngoscope.

[42] Noble W., Tyler R., Dunn C., Bhullar N. (2008). Unilateral and bilateral cochlear implants and the implant-plus-hearing-aid profile: Comparing self-assessed and measured abilities. Int. J. Audiol..

[43] Gifford R.H., Shallop J.K., Peterson A.M. (2008). Speech recognition materials and ceiling effects: Considerations for cochlear implant programs. Audiol. Neurootol..

